# Construction of ultrasonic nanobubbles carrying CAIX polypeptides to target carcinoma cells derived from various organs

**DOI:** 10.1186/s12951-017-0307-0

**Published:** 2017-09-29

**Authors:** Lianhua Zhu, Yanli Guo, Luofu Wang, Xiaozhou Fan, Xingyu Xiong, Kejing Fang, Dan Xu

**Affiliations:** 1 Department of Ultrasound, Southwest Hospital, Third Military Medical University, 30 Gaotanyan Street, Shapingba District, Chongqing, 400038 China; 2 Department of Urology, Daping Hospital, Third Military Medical University, 10 Changjiang Zhi Road, Yuzhong District, Chongqing, 400038 China

**Keywords:** Ultrasound molecular imaging, Targeted nanobubbles, Carbonic anhydrase IX, Malignant tumors

## Abstract

**Background:**

Ultrasound molecular imaging is a novel diagnostic approach for tumors, whose key link is the construction of targeted ultrasound contrast agents. However, available targeted ultrasound contrast agents for molecular imaging of tumors are only achieving imaging in blood pool or one type tumor. No targeted ultrasound contrast agents have realized targeted ultrasound molecular imaging of tumor parenchymal cells in a variety of solid tumors so far. Carbonic anhydrase IX (CAIX) is highly expressed on cell membranes of various malignant solid tumors, so it’s a good target for ultrasound molecular imaging. Here, targeted nanobubbles carrying CAIX polypeptides for targeted binding to a variety of malignant tumors were constructed, and targeted binding ability and ultrasound imaging effect in different types of tumors were evaluated.

**Results:**

The mean diameter of lipid targeted nanobubbles was (503.7 ± 78.47) nm, and the polypeptides evenly distributed on the surfaces of targeted nanobubbles, which possessed the advantages of homogenous particle size, high stability, and good safety. Targeted nanobubbles could gather around CAIX-positive cells (786-O and Hela cells), while they cannot gather around CAIX-negative cells (BxPC-3 cells) in vitro, and the affinity of targeted nanobubbles to CAIX-positive cells were significantly higher than that to CAIX-negative cells (P < 0.05). Peak intensity and duration time of targeted nanobubbles and blank nanobubbles were different in CAIX-positive transplanted tumor tissues in vivo (P < 0.05). Moreover, targeted nanobubbles in CAIX-positive transplanted tumor tissues produced higher peak intensity and longer duration time than those in CAIX-negative transplanted tumor tissues (P < 0.05). Finally, immunofluorescence not only confirmed targeted nanobubbles could pass through blood vessels to enter in tumor tissue spaces, but also clarified imaging differences of targeted nanobubbles in different types of transplanted tumor tissues.

**Conclusions:**

Targeted nanobubbles carrying CAIX polypeptides can specifically enhance ultrasound imaging in CAIX-positive transplanted tumor tissues and could potentially be used in early diagnosis of a variety of solid tumors derived from various organs.

**Electronic supplementary material:**

The online version of this article (doi:10.1186/s12951-017-0307-0) contains supplementary material, which is available to authorized users.

## Background

Early diagnosis and timely treatment of malignant tumors can increase the survival and decrease the morbidity of patients. Compared to contrast-enhanced magnetic resonance imaging (CE-MRI) and contrast-enhanced computed tomography (CE-CT), contrast-enhanced ultrasound (CEUS) has the advantages of easy operation, no radiation and real-time dynamic imaging. Therefore, it has rapidly become popular in clinical application [1]. With the continuous development of CEUS, ultrasound molecular imaging has emerged. Constructing ultrasound contrast agents (UCAs) is the key link in ultrasound molecular imaging. Studies have already confirmed that targeted UCAs carrying specific antibodies or ligands can bind specifically to tissues or disease lesions that express specific targets. These agents specifically aggregate in target tissues through the blood circulation, thus achieving targeted ultrasound imaging at the molecular or cellular level [2–4].

Because tumor neovascularization has incomplete basement membranes, lack smooth muscle layer, and blood vessel wall gaps are 380–780 nm, which are much more permeable than vessels in normal tissues, particles smaller than 700 nm in diameter can theoretically pass through tumor blood vessels and enter into tumor tissue spaces [5]. However, because the size of UCAs currently used is 2–8 µm, they only can be used for imaging in blood pool and cannot pass through tumor blood vessels to achieve ultrasound molecular imaging of parenchymal cells in tumor tissues [6]. Due to enhanced permeability and retention (EPR) effect, some studies have shown that nanoscale UCAs with the particle size smaller than 700 nm can be used to achieve targeted ultrasound molecular imaging of tumor parenchyma tissues [7]. Nanobubbles containing a core gas coated with a lipid membrane are a type of nanoscale UCAs. Due to the same contrast enhanced effect as routine clinical UCAs, nanobubbles have broad prospects for clinical application [8–10]. Our previous studies have constructed targeted nanobubbles that specifically target prostate cancer. Imaging duration time and peak intensity provided by these nanobubbles in transplanted tumors were significantly better than those of blank nanobubbles, which could be used for achieving specific targeted imaging of prostate cancer and providing the research basis and methods for targeted molecular imaging of tumors [11–13].

However, our previously constructed targeted nanobubbles had significant limitations. For example, they could only be used in targeted ultrasound imaging of prostate cancer and not in targeted imaging of other tumors, so the tumor type to be selected was limited. Carbonic anhydrase IX (CAIX) is highly expressed on cell membranes of many malignant solid tumors and is not expressed in most normal tissues, except gastrointestinal tracts and biliary tracts. These features are conducive to specific binding between targeted UCAs carrying anti-CAIX ligands and tumor cells and therefore provide an ideal target for ultrasound molecular imaging of a variety of tumors, including kidney cancer and cervical cancer [14–17].

Polypeptides consist of α-amino acids linked each other through peptide bonds. The chemical structure of polypeptides is simple, their molecular weight is small, and their properties are stable, so they have been used extensively in medical diagnosis and drug development [18–21]. Askoxylakis et al. used the phage display technology to screen polypeptides PGLR-P1 (NMPKDVTTRMSS) against proteoglycan domain of CAIX protein, which is an ideal target ligand of CAIX [22]. Therefore, based on the aforementioned study, we constructed targeted nanobubbles that carry CAIX polypeptides (PGLR-P1) and then tested their safety and relevant physical properties. In addition, the imaging effect of targeted nanobubbles carrying CAIX polypeptides in many CAIX-positive tumors was investigated in vitro and in vivo.

## Methods

### Synthesis and validation of the biotinylated polypeptides

The biotinylated polypeptides PLGR-P1 (NMPKDVTTRMSSk-biotin) and the biotinylated polypeptides modified with FITC were synthesized using chemical solid-phase synthesis method. The biotinylated polypeptides were synthesized according to the procedures described below: after resin (Nankai Synthesis Technology Co. Ltd, Tianjin, China) swelling by dimethyl formamide (Tianma Medical Fine Chemicals Co. Ltd, Suzhou, China), Fmoc-lysine (Tianma Medical Fine Chemicals Co. Ltd) was incorporated. Then fmoc protection was removed, and amino acids on the resin were detected using ninhydrin (Tianma Medical Fine Chemicals Co. Ltd). Above steps were repeated for each successive amino acid in sequence. At last, the polypeptides were modified with biotin (Tianma Medical Fine Chemicals Co. Ltd). After the synthesis was completed, the synthetic polypeptides were separated and purified by a LC-10ATVP high-performance liquid chromatograph (SHIMADZU, Kyoto, Japan). A LCMS-8030 electrospray ionization mass spectrometer (SHIMADZU) was used to perform mass spectrometry on the pure polypeptides. Some pure biotinylated polypeptides were modified with FITC (Tianma Medical Fine Chemicals Co. Ltd).

### Construction and validation of targeted nanobubbles

A total of 11 mg of 2-dipalmitoyl-sn-glycero-3-phosphocholine (Avanti Polar Lipids, Alabaster, AL, USA), 1,2-dipalmitoyl-sn-glycero-3-phosphoglycerol (Corden Pharma, Liestal, Switzerland), 1,2-dipalmitoyl-sn-glycero-3-phosphoethanolamine (Corden Pharma), 1,2-dipalmitoyl-sn-glycero-3-phosphatidic acid (Corden Pharma), and biotin-modified 1,2-distearoyl-sn-glycero-3-phosphoethanolamine (NANOCS, Boston, MA, USA) lipid mixture at a certain ratio was dissolved in a mixture solution containing phosphate-buffered saline (PBS) and glycerin mixture, then oscillated overnight in a SHKE420HP orbital shaker (Thermo, Waltham, MA, USA), and transferred to a penicillin bottle. The air in the bottle was replaced with C_3_F_8_ (Research Institute of Physical and Chemical Engineering of Nuclear Industry, Tianjin, China), and the bottle was oscillated for 80 s using a ST-B series amalgamator (AT&M Biomaterials Co., Beijing, China). The sample was transferred to a 10-ml centrifuge tube and centrifuged to remove insoluble lipids. After the sample was mixed evenly and centrifuged again, nanobubbles were obtained. Added streptavidin (Solarbio Science & Technology Co. Ltd, Beijing, China) at a ratio of 3 µg streptavidin/10^7^ nanobubbles, and the mixture was incubated at 4 °C for 1 h. After the sample was washed three times, the biotinylated polypeptides were added in a proper ratio, and the mixture was incubated at 4 °C for 1 h. The mixture was then washed three times, so targeted nanobubbles (TN) carrying CAIX polypeptides were obtained. Blank nanobubbles (BN) that did not carry CAIX polypeptides were used as a control. To directly prove that biotin–streptavidin system could stably and efficiently link the biotinylated polypeptides to the surfaces of targeted nanobubbles, we used the same method to link the FITC-modified biotinylated polypeptides to the surfaces of targeted nanobubbles and used DiI dye (Beyotime Institute of Biotechnology, Shanghai, China) to label targeted nanobubbles lipid membranes, then observed fluorescence intensity of targeted nanobubbles under a Zeiss 780 laser scanning confocal microscope (LSCM) (Carl Zeiss AG, Oberkochen, Germany).

### Cell culture in vitro

Three commonly used cell lines, including 786-O (human kidney carcinoma cells, CAIX-positive cells), Hela (human cervical carcinoma cells, CAIX-positive cells) and BxPC-3 (human pancreatic carcinoma cells, CAIX-negative cells) cells, were used in the study. All cell lines were purchased from the American Type Culture Collection (ATCC, Manassas, VA, USA). The complete medium was made of RPMI 1640 culture medium supplemented with 10% fetal bovine serum, 100 IU/ml penicillin, and 100 µg/ml streptomycin. The cells were cultured in an incubator at 37 °C and in 5% CO_2_.

### Determination of basic characteristics of targeted nanobubbles

The concentrations of targeted and blank nanobubbles were measured using a hemocytometer. The particle size and zeta potential of targeted nanobubbles and blank nanobubbles were measured by Malvern Zetasizer nano ZS90 detector (Malvern Instruments Inc., Worcestershire, UK). All measurements were performed three times. The distribution of targeted nanobubbles was observed under an IX71 optical microscope (Olympus Corporation, Kyoto, Japan), and their morphology was observed under a JEM-1400 transmission electron microscope (JEOL, Tokyo, Japan). To evaluate the cytotoxicity of targeted nanobubbles on 786-O cells, MTT assay was performed. 786-O cells at the logarithmic growth phase were inoculated into a 96-well plate at the concentration of 10^4^/well. After culturing overnight, various concentrations of targeted and blank nanobubbles were added. Freshly prepared MTT solution (Beyotime Institute of Biotechnology) was added after the cells had been cultured with nanobubbles for 4 h, then 100 µl dimethyl sulphoxide (DMSO) was added. OD_550_ values were measured using a Multiskan Spectrum (Thermo) to compare the cytotoxicity of targeted and blank nanobubbles. The changes in the concentration and particle size of targeted nanobubbles over time were evaluated. Targeted nanobubbles were kept at 4 °C, and measured at 0, 1, 2, 4, 7 days.

### In vitro binding experiments with targeted nanobubbles

Logarithmic-phase 786-O, HeLa, and BxPC-3 cells were inoculated into a 24-well plate at the concentration of 10^5^/well. After culturing overnight, the cells were fixed in 4% paraformaldehyde and blocked in PBS with 5% bovine serum albumin (BSA-PBS). Each type of cells was divided into three groups. The 1st group received 30 µl of targeted nanobubbles at the concentration of 2 × 10^7^/ml, the 2nd group received 30 µl of the equal concentration of blank nanobubbles, and the 3rd group pre-blocked with 100 µl of the polypeptides solution at the concentration of 1 mg/ml for 2 h received 30 µl of the equal concentration of targeted nanobubbles. Then the cells were incubated with nanobubbles at 4 °C for 2 h. Slides were washed three times and placed upside down. The number of nanobubbles binding to these three types of cells was observed under an optical microscope. The affinity of targeted nanobubbles to the cells was performed using a FACSCalibur flow cytometer (BD Biosciences, San Jose, CA, USA) [23, 24]. The tumor cells at the logarithmic growth phase were dissociated and collected into Eppendorf tubes at the concentration of 5 × 10^5^/tube. Each type of cells was divided into three groups. The 1st group served as a control was mixed with only the cells, the 2nd group was mixed the cells with 500 µl of blank nanobubbles at the concentration of 2 × 10^7^/ml, and the 3rd group was mixed the cells with 500 µl of the equal concentration of targeted nanobubbles. The mixtures were then incubated at 37 °C for 40 min. The supernatant was removed after centrifugation. Then the cells were re-suspended in 250 µl PBS, and measured the affinity of targeted and blank nanobubbles to the cells by flow cytometry.

### In vitro imaging experiments with targeted nanobubbles

A model for in vitro imaging of targeted nanobubbles was prepared using 1% agarose powder. IU22 ultrasound diagnosis apparatus and 50-mm linear probe (Philips, Amsterdam, Netherlands) were used for imaging. After the probe was fixed, imaging mode was adjusted (the frequency was 5–12 MHz, the mechanical index was 0.12, and the gain was 85%). The imaging intensity of targeted and blank nanobubbles at five different concentrations (1.0 × 10^8^, 5.0 × 10^7^, 2.5 × 10^7^, 1.0 × 10^7^, and 5.0 × 10^6^/ml) and the decay rate of them at the concentration of 5 × 10^7^/ml were compared in vitro. In addition, the imaging intensity of targeted nanobubbles at the concentration of 5 × 10^7^/ml was compared before and after destruction by ultrasound waves with the high mechanical index.

### In vivo imaging experiments with targeted nanobubbles

The study was approved by the Laboratory Animal Welfare and Ethics Committee of the Third Military Medical University and was performed in accordance with the NIH Guide for the Care and Use of Laboratory Animals. Logarithmic-phase 786-O and HeLa cells at the concentration of 5 × 10^7^/ml and logarithmic-phase BxPC-3 cells at the concentration of 1 × 10^7^/ml were implanted subcutaneously on both sides of 4-week-old BALB/c-nu nude mice back (HFK Bioscience Co., Beijing, China). When the volume of the subcutaneous tumor in tumor-bearing nude mice reached approximately 1 cm^3^, five 786-O tumor-bearing nude mice, five Hela tumor-bearing nude mice and five BxPC-3 tumor-bearing nude mice were randomly collected and were anesthetized by intraperitoneal injection of 1% pentobarbital sodium. Ultrasound imaging was performed using IU22 ultrasound diagnosis apparatus and 50-mm linear probe. The surfaces of the transplanted tumors were covered with coupling agents. When the probe clearly displayed the largest cross-section of the transplanted tumor, the probe was fixed. After the 2-dimensional grayscale was collected, the imaging mode was adjusted to contrast-enhanced imaging mode (the frequency was 5–12 MHz, the mechanics index was 0.12, and the gain was 85%), and the focus center was located at the center of the transplanted tumor tissues. 200 µl of blank nanobubbles or targeted nanobubbles at the same concentration were injected into each nude mouse through the tail vein at random, and the tubing was washed with 100 µl of normal saline. The images were continuously and dynamically collected. After 1 h, nanobubbles in nude mice bodies completely disappeared, the same method was used to inject 200 µl of nanobubbles suspension containing the same concentration of the other nanobubbles, which guaranteed nanobubbles would not interfere with the imaging effect of each other. The same process was used to collect ultrasound images of targeted and blank nanobubbles in liver and kidney tissues. The collected images were analyzed using Qlab8.1 software (Philips). Three indicators (peak time, peak intensity, and duration time) were compared for targeted and blank nanobubbles in liver and kidney, CAIX-positive and CAIX-negative transplanted tumor tissues. Peak time referred to the time interval between the injection of nanobubbles and the time at which peak intensity was reached. Duration time referred to the time interval between the injection of nanobubbles and the completion of enhanced imaging.

### Immunohistochemistry identification

The tumor-bearing nude mice were divided into two groups. After the completion of ultrasound contrast imaging, the nude mice in one group were injected with 200 µl of DiI-labeled targeted nanobubbles through the tail vein. When peak intensity was achieved, the transplanted tumor tissues and the right thigh muscle tissues were collected and stored at −20 °C. The frozen specimens were placed on tissue holder, then added frozen embedding medium to solidify the specimens. After fixed in 4% paraformaldehyde, the sections were incubated with rat anti-mouse CD31 monoclonal antibody (Abcam, Cambridge, MA, USA) (1:300 dilution) at 4 °C overnight. FITC-labeled rabbit anti-rat secondary antibody (Beyotime Institute of Biotechnology) was added, and the sections were incubated for 2 h at room temperature in the dark. After washing with PBS, the sections were counterstained with DAPI for 10 min, and observed under a LSCM. To further clarify the targeting ability of targeted nanobubbles, the expression of CD31 and CAIX were detected by immunohistochemical staining in paraffin sections. After the completion of ultrasound contrast imaging, the other mice were sacrificed and the transplanted tumor tissues were collected. Then the tumor tissues were fixed in paraformaldehyde, embedded in paraffin and sectioned. After routine deparaffination and blocking in 3% H_2_O_2_, the sections were incubated with rabbit anti-mouse CD31 polyclonal antibody (Abcam) (1:100 dilution) or rabbit anti-human CAIX monoclonal antibody (Abcam) (1:400 dilution) at 4 °C overnight. Next, horseradish peroxidase-labeled goat anti-rabbit secondary antibody (Beyotime Institute of Biotechnology) was added, and the sections were incubated at 37 °C for 1 h. After in the presence of DAB reagent and counterstaining cell nuclei with hematoxylin, the sections were mounted and examined under an optical microscope. Microvessel density (MVD) was determined from five random fields (at × 200 magnification) of each section, and only tumor blood vessels with vascular lumen or linear vascular shape were used for MVD analysis [25].

### Statistical analysis

The Statistical Package for Social Sciences 22.0 (IBM Corporation, Armonk, NY, USA) was used for data analysis. All quantitative data are expressed as the mean ± standard deviation. All parameter indicators were examined using paired t test and one-way analysis of variance. P < 0.05 indicated statistical significance. Histograms were plotted using GraphPad Prism 6.0 (GraphPad Software, Inc., La Jolla, CA, USA).

## Results

### Synthesis and purification of polypeptides and FITC-modified polypeptides

The polypeptides and the FITC-modified polypeptides were analyzed by high performance liquid chromatography, and both their purity was greater than 95%. The relative molecular weight of the biotinylated polypeptide detected by mass spectrometry was 1722.2 ± 0.4, the theoretically calculated molecular weight of the biotinylated polypeptide was 1721.1 [see Additional file 1a]. The relative molecular weight of the FITC-modified biotinylated polypeptides was 2223.8 ± 0.6, the theoretically calculated molecular weight of the FITC-modified polypeptides was 2223.6 [see Additional file 1b]. Therefore, the relative molecular weight of the synthesis polypeptides and the synthesis FITC-modified polypeptides were consistent with the theoretically calculated molecular weight.

### Construction and identification of targeted nanobubbles

**Fig. 1:**
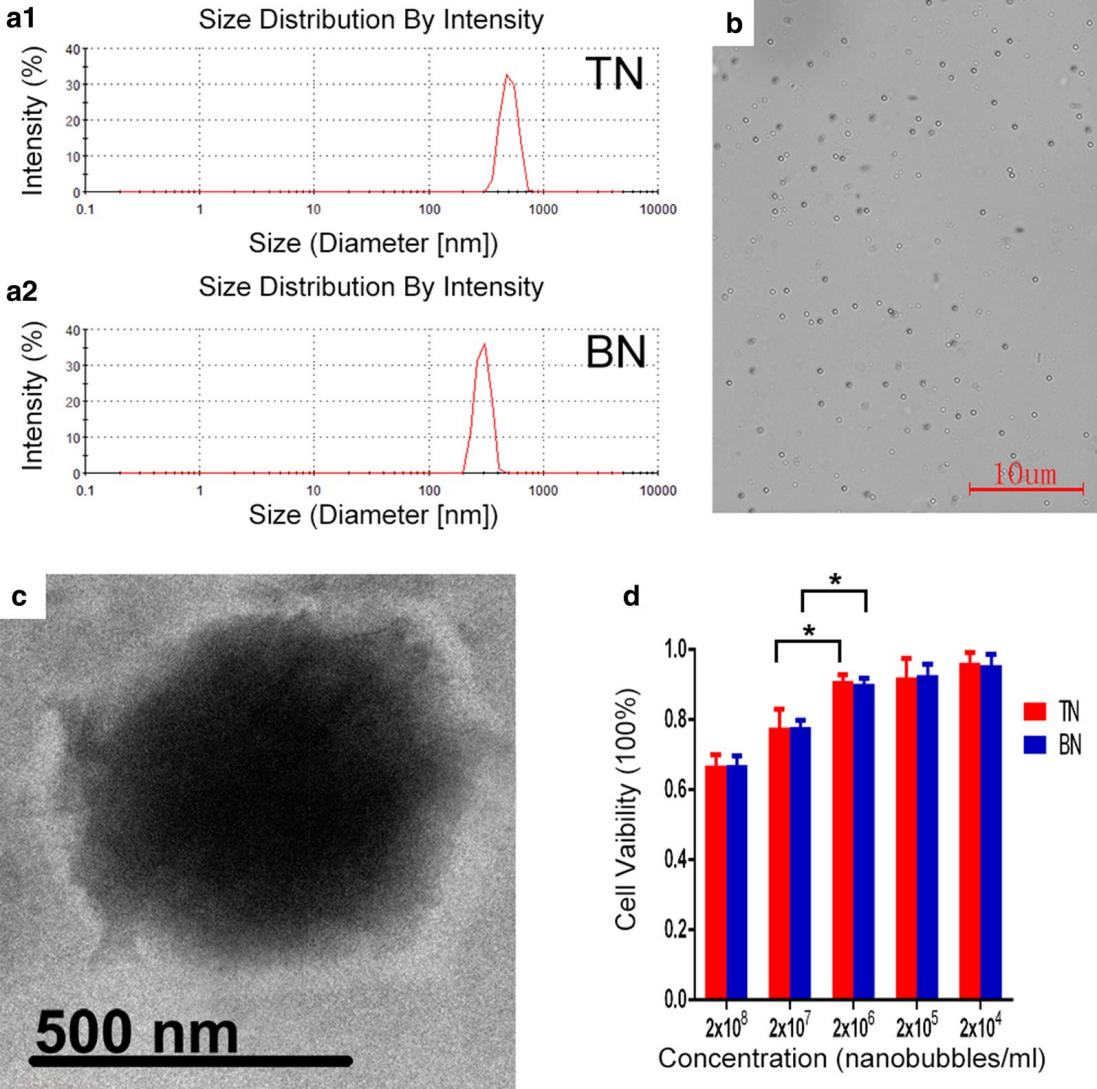
Basic characteristics of targeted nanobubbles. **a1** The particle size of targeted nanobubbles. **a2** The particle size of blank nanobubbles. **b** The distribution of targeted nanobubbles under an optical microscope. **c** The morphology of targeted nanobubbles under a transmission electron microscope. **d** MTT assay of targeted and blank nanobubbles. *P < 0.05

The concentrations of targeted and blank nanobubbles were (10.70 ± 0.82) × 10^8^/ml and (11.53 ± 0.81) × 10^8^/ml, which were not significantly different (P > 0.05). The particle sizes of targeted nanobubbles and blank nanobubbles determined with a Malvern Zetasizer nano ZS90 analyzer were 503.7 ± 78.5 nm and 428.1 ± 41.8 nm, respectively (Fig. 1a). The zeta potential of blank nanobubbles was −19.27 ± 2.21 mV. The negative zeta potential of blank nanobubbles may be mainly ascribed to 1,2-dipalmitoyl-sn-glycero-3-phosphatidic acid, which is an anionic phospholipid [26]. When targeted nanobubbles were loaded with CAIX polypeptides, the zeta potential increased to −14.80 ± 2.12 mV, implying that the positively charged amino groups on CAIX polypeptides neutralized partial negative charge of anionic phospholipid [27]. Negative charge could keep the stability of targeted nanobubbles through the electrostatic repulsion. Under an optical microscope, targeted nanobubbles appeared evenly distributed, had similar particle sizes, did not display obvious aggregation or rupture, and showed good stability (Fig. 1b). Viewed by a transmission electron microscopy, they appeared regular and round in shape, with smooth boundaries, and the particle size was approximately 500 nm (Fig. 1c), which was consistent with the measurement results obtained with a Malvern Zetasizer nano ZS90 analyzer. The cytotoxicity of targeted and blank nanobubbles on 786-O cells was measured by MTT assay, which showed that the cytotoxicity of targeted nanobubbles and blank nanobubbles at the same concentrations was not significantly different (P > 0.05), indicating that bound polypeptides did not significantly alter the cytotoxicity of nanobubbles (Fig. 1d). When the concentration of targeted nanobubbles at 2 × 10^7^/ml, the survival rate of the cells (77.76% ± 1.87%) was significantly lower than that (90.23% ± 1.52%) observed at the concentration of 2 × 10^6^/ml (P < 0.05). The survival rate of 786-O cells was also different when the concentrations of blank nanobubbles were 2 × 10^7^ and 2 × 10^6^/ml (P < 0.05).

**Fig. 2:**
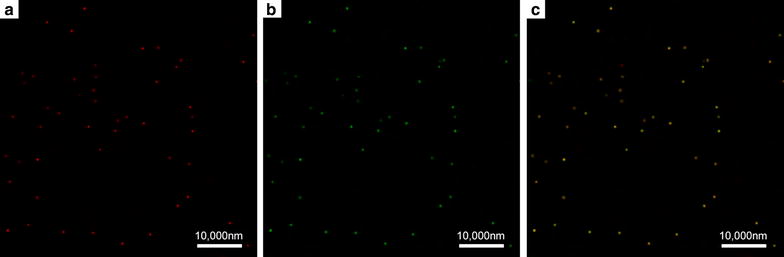
Dual-fluorescence validates targeted nanobubbles. **a** DiI-labeled targeted nanobubbles. **b** Nanobubbles carrying the FITC-modified polypeptides. **c** Merged image confirms that biotin–streptavidin system successfully linked the polypeptides to the surfaces of targeted nanobubbles

Under a LSCM, lipid membrane of DiI-labeled targeted nanobubbles displayed red fluorescence (Fig. 2a), whereas the surfaces of targeted nanobubbles carrying the FITC-modified polypeptides displayed green fluorescence (Fig. 2b). These two types of fluorescence could overlap completely (Fig. 2c). Blank nanobubbles that did not carry the polypeptides and targeted nanobubbles that carried the unmodified polypeptides did not display green fluorescence.

**Fig. 3:**
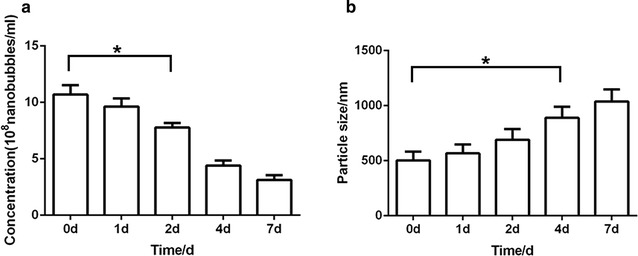
Stability of targeted nanobubbles. **a** Histogram of the concentration of targeted nanobubbles changes over time. **b** Histogram of the particle size of targeted nanobubbles changes over time. *P < 0.05

The concentration of targeted nanobubbles decreased stored at 4 °C, and were (9.64 ± 0.72) × 10^8^, (7.72 ± 0.40) × 10^8^, (4.40 ± 0.45) × 10^8^, (3.11 ± 0.44) × 10^8^/ml at 1, 2, 4, 7 days, respectively (Fig. 3a). After 2 days, the concentration of targeted nanobubbles was different from that at 0 days (P < 0.05). The particle size of targeted nanobubbles became larger over time, and were 567.2 ± 80.4, 690.8 ± 95.5, 889.6 ± 99.7, 1038 ± 109.6 nm after stored for 1, 2, 4, 7 days, respectively (Fig. 3b). After 4 days of storage, the particle size of targeted nanobubbles was different from that at 0 days (P < 0.05).

### In vitro specific binding experiments with targeted nanobubbles

**Fig. 4:**
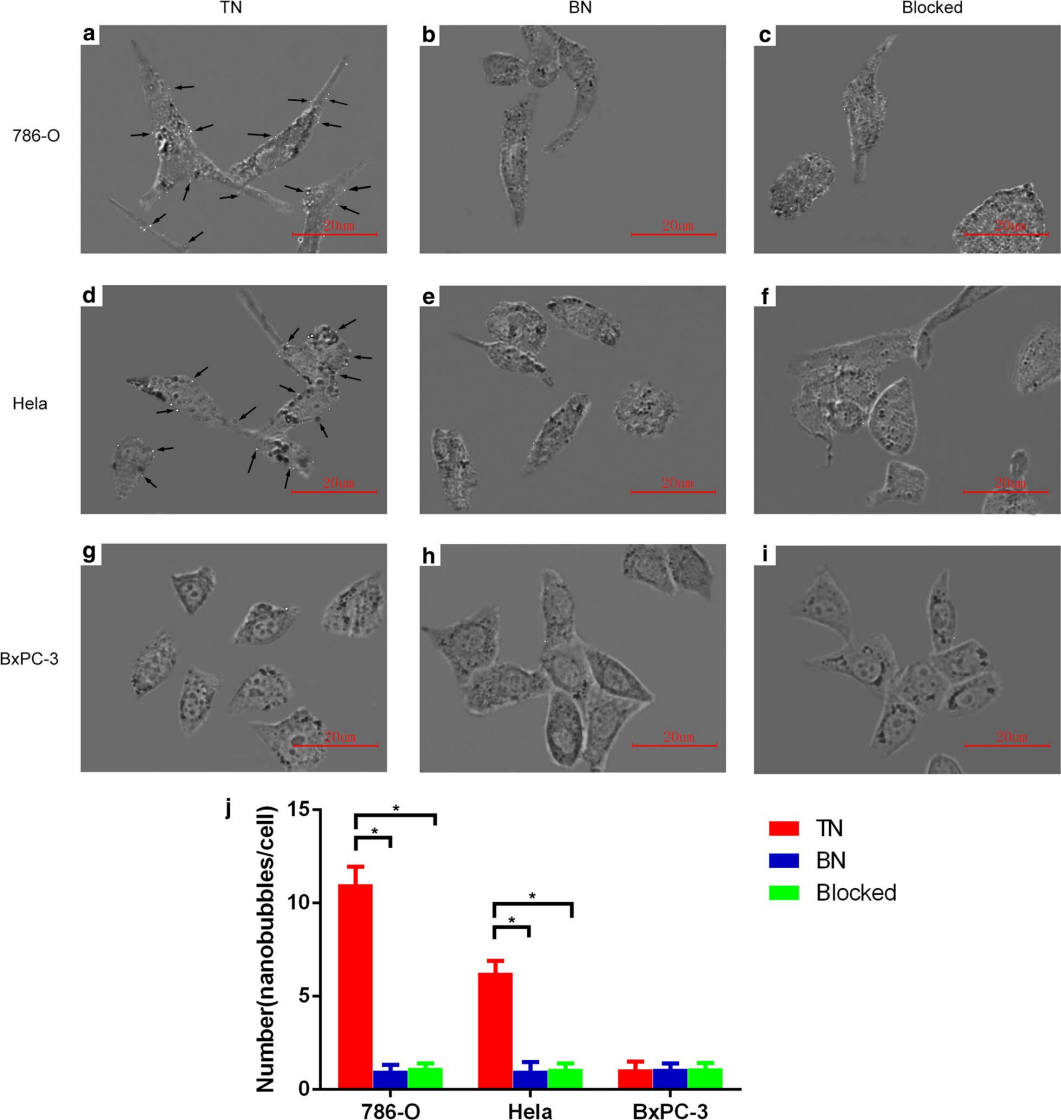
In vitro targeting experiments of targeted and blank nanobubbles. **a**–**c** The binding of nanobubbles (black arrows) to 786-O cells. **d**–**f** The binding of nanobubbles (black arrows) to Hela cells. **g**–**i** The binding of nanobubbles to BxPC-3 cells. **j** The number of nanobubbles binding to the three types of tumor cells. *P < 0.05

The binding of targeted nanobubbles to the three types of cells was observed under an optical microscope (Fig. 4). The number of targeted nanobubbles binding to 786-O cells was significantly higher than that of blank nanobubbles binding to the cells, also higher than that of targeted nanobubbles binding to the cells pre-blocked by the polypeptides (Fig. 4a–c). The number of targeted nanobubbles binding to Hela cells was significantly higher than that of blank nanobubbles binding to the cells, also higher than that of targeted nanobubbles binding to the cells pre-blocked by the polypeptides (Fig. 4d–f). The number of nanobubbles binding to BxPC-3 cells did not differ significantly in all groups (Fig. 4g–i). Figure 4j showed the number of targeted nanobubbles and blank nanobubbles binding to the three types of tumor cells.

**Fig. 5:**
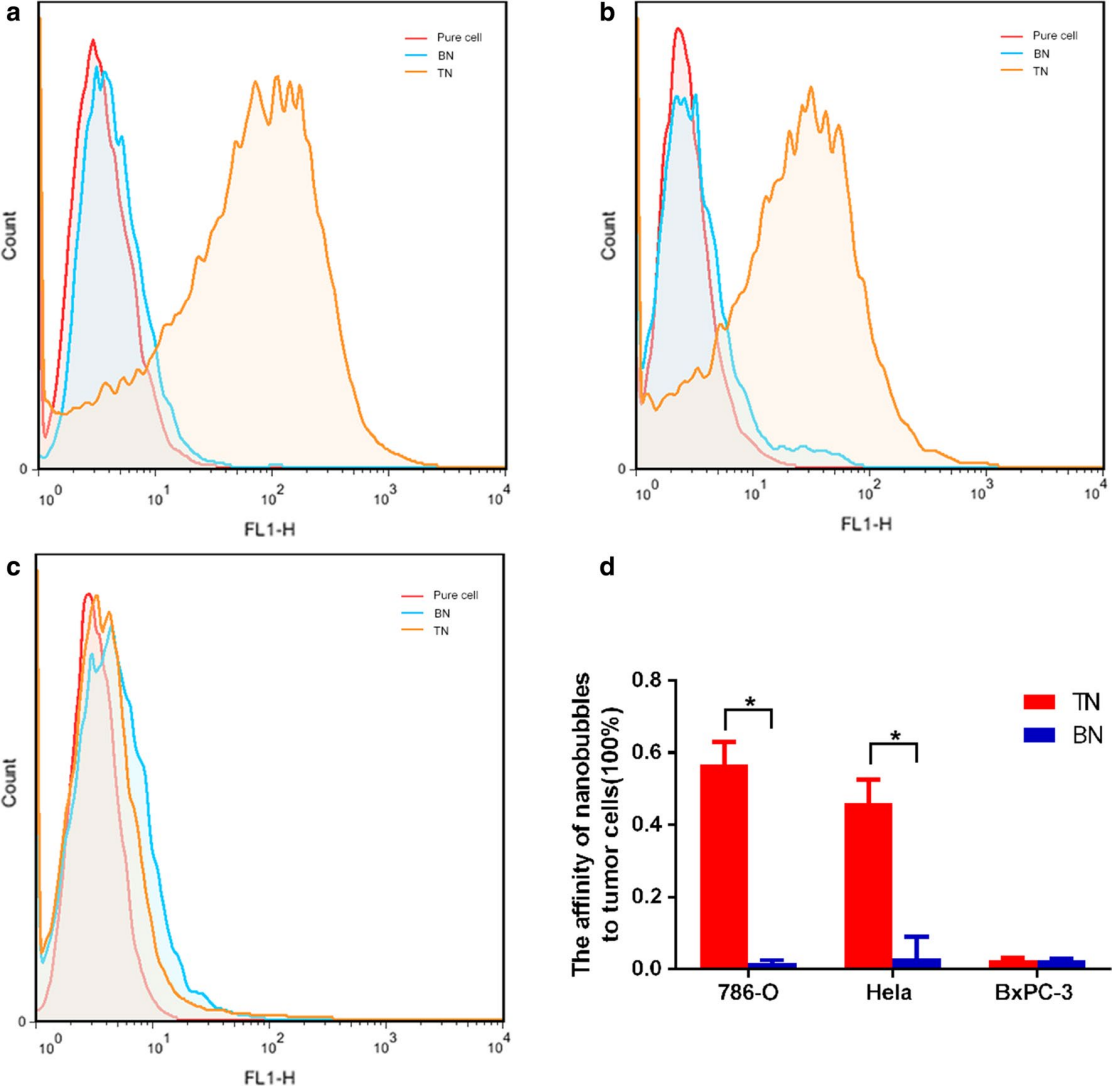
Affinity of targeted and blank nanobubbles to three types of cells. **a** The affinity of nanobubbles to 786-O cells. **b** The affinity of nanobubbles to HeLa cells. **c** The affinity of nanobubbles to BxPC-3 cells. **d** Quantification of the affinity of the two types of nanobubbles to the three types of cells. *P < 0.05

The affinity of targeted nanobubbles and blank nanobubbles to the three types of cells was determined by flow cytometry. The affinity of targeted nanobubbles to 786-O cells and HeLa cells was significantly higher than that of blank nanobubbles to the two types of cells (Fig. 5a, b), whereas the affinity of the two types of nanobubbles to BxPC-3 cells did not differ significantly (Fig. 5c). Quantification of the affinity of targeted nanobubbles and blank nanobubbles to the three types of cells was shown in Fig. 5d. These results were consistent with the results obtained with an optical microscopy.

### In vitro imaging experiments with targeted nanobubbles

**Fig. 6:**
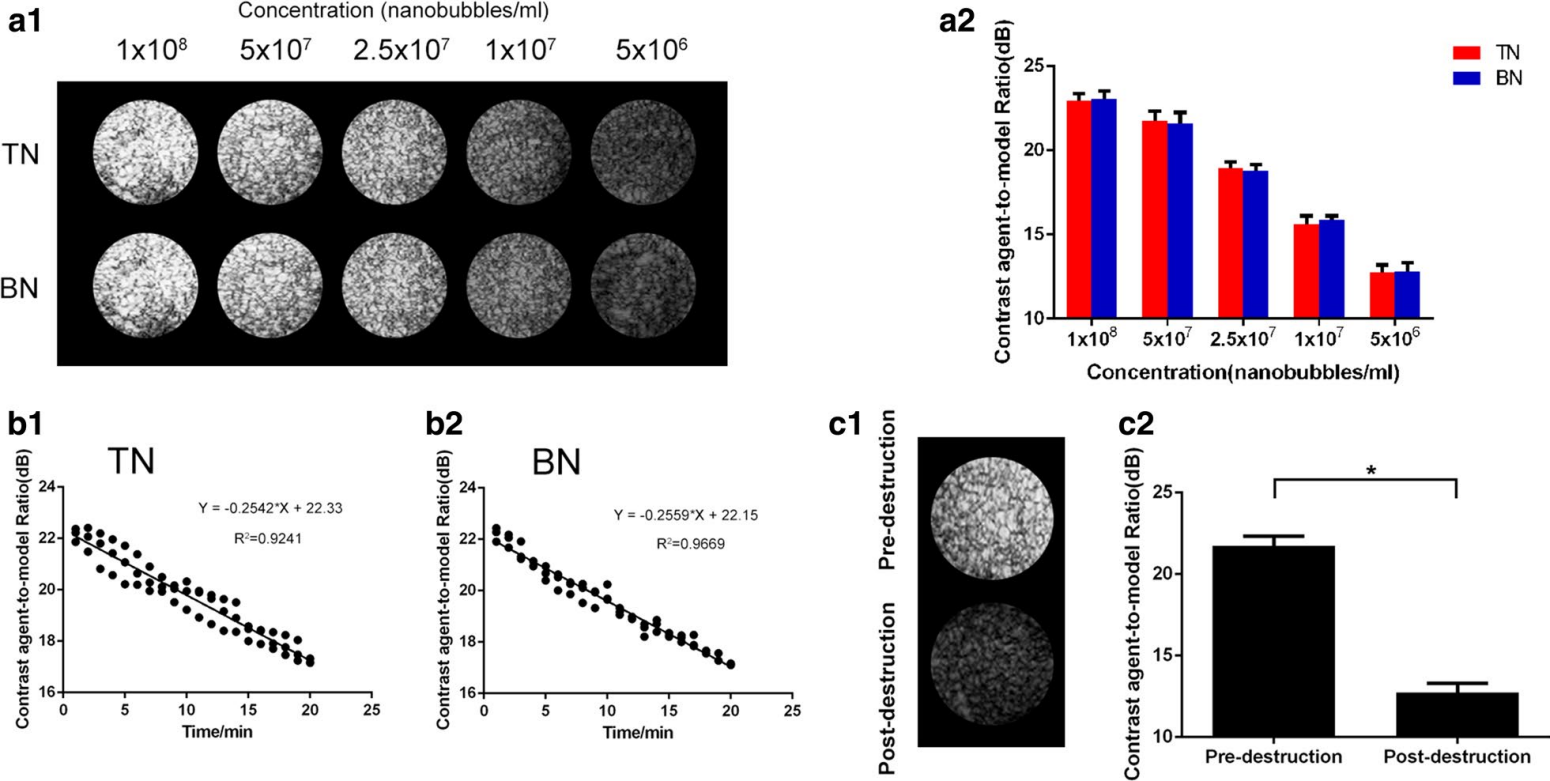
In vitro imaging of targeted and blank nanobubbles. **a1** Ultrasound images of nanobubbles at different concentrations in vitro. **a2** Quantification of imaging intensity of nanobubbles in vitro. **b1** The decay rate of targeted nanobubbles in vitro. **b2** The decay rate of blank nanobubbles in vitro. **c1** Ultrasound images of targeted nanobubbles before and after destruction. **c2** Quantification of imaging intensity of targeted nanobubbles before and after destruction. *P < 0.05

In in vitro imaging model, the imaging intensity of targeted and blank nanobubbles at various concentration gradients did not show a significant difference (Fig. 6a). During 20 min of continuous and dynamic imaging in in vitro model, the imaging intensity of targeted and blank nanobubbles decreased over time. Linear fitting of the imaging intensity at different time points, taking the slope of the line as the decay rate of nanobubbles, the decay rate of targeted nanobubbles and blank nanobubbles was not significantly different [28] (P > 0.05) (Fig. 6b). After the “manual flash” technique was used, the imaging intensity of targeted nanobubbles was significantly decreased (Fig. 6c), indicating that targeted nanobubbles could be destructed by ultrasound waves with the high mechanical index.

### In vivo imaging experiments with targeted nanobubbles

**Fig. 7:**
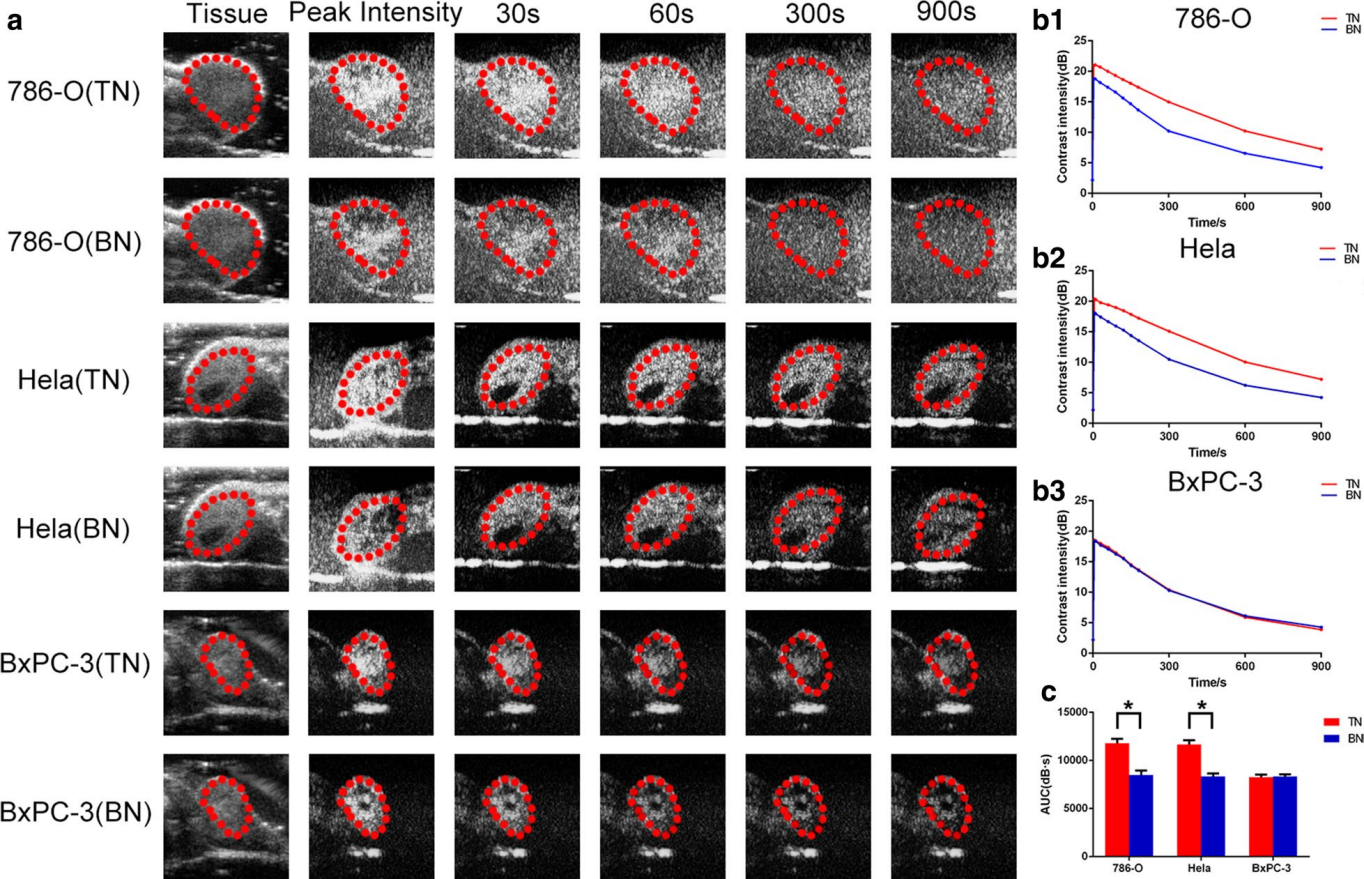
Imaging characteristics of targeted and blank nanobubbles in three types of transplanted tumor tissues. **a** Ultrasound images of nanobubbles in the three types of transplanted tumor tissues. Red circles indicate the regions of transplanted tumor tissues. **b1**–**b3** Time-intensity curves of nanobubbles in the three types of transplanted tumor tissues. **c** Area under the curve of nanobubbles in the three types of transplanted tumor tissues. *P < 0.05

Targeted and blank nanobubbles were used to image liver, kidney and the three types of transplanted tumor tissues in nude mice. Peak time, peak intensity, and duration time of targeted nanobubbles and blank nanobubbles had no significant difference in liver, kidney, and BxPC-3 transplanted tumor tissues (P > 0.05) (Table 1). In 786-O and HeLa transplanted tumor tissues, peak time of the two types of nanobubbles also had no significant difference (P > 0.05). However, peak intensity of targeted nanobubbles was significantly higher than that of blank nanobubbles (P < 0.05). In addition, duration time of targeted nanobubbles was also significantly longer than that of blank nanobubbles (P < 0.05) (Table 1 and Fig. 7a). Peak intensity and duration time of targeted nanobubbles in 786-O and HeLa transplanted tumor tissues were also significantly superior to those in BxPC-3 transplanted tumor tissues (P < 0.05) (Table 1 and Fig. 7a). Dynamic analysis of the time-intensity curves of ultrasound imaging in the three types of transplanted tumor tissues from 0 to 15 min, the imaging intensity of targeted nanobubbles was always higher than that of blank nanobubbles in 786-O and HeLa transplanted tumor tissues (P < 0.05), while the imaging intensity of targeted nanobubbles was not different from blank nanobubbles in BxPC-3 transplanted tumor tissues (P > 0.05) (Fig. 7b). Moreover, in 786-O and Hela transplanted tumor tissues, the difference of imaging intensity between targeted nanobubbles and blank nanobubbles increased over time from 0 min to 5 min, while the difference decreased over time from 5 min to 15 min. In 786-O and HeLa transplanted tumor tissues, area under the curve of targeted nanobubbles was significantly larger than that of blank nanobubbles (11792.49 ± 453.32 vs 8498.74 ± 449.75 and 11658.93 ± 431.65 vs 8348.65 ± 289.44), while area under the curve of targeted nanobubbles was not significantly different from blank nanobubbles in BxPC-3 transplanted tumor tissues (8262.62 ± 267.28 vs 8328.89 ± 209.33) (Fig. 7c).

**Table 1:** Ultrasound parameters of targeted and blank nanobubbles in liver, kidney and transplanted tumors

Tissue	Contrast agent	Peak time/s	Peak intensity/dB	Duration time/min
Liver	TN	6.22 ± 0.50	18.31 ± 0.61	21.46 ± 0.87
	BN	6.32 ± 0.95	18.52 ± 0.95	21.19 ± 1.68
Kidney	TN	2.01 ± 0.34	17.48 ± 0.76	20.11 ± 1.20
	BN	2.37 ± 0.59	17.42 ± 0.87	19.77 ± 0.77
786-O tumor	TN	8.28 ± 0.74	21.56 ± 0.62*^,#^	21.35 ± 0.51*^,#^
	BN	8.44 ± 0.71	19.30 ± 0.68	17.91 ± 0.81
Hela tumor	TN	8.31 ± 0.75	20.51 ± 0.69*^,#^	20.93 ± 0.61*^,#^
	BN	8.25 ± 0.78	18.53 ± 0.42	17.99 ± 0.85
BxPC-3 tumor	TN	8.04 ± 0.85	18.34 ± 0.65	17.55 ± 0.53
	BN	8.41 ± 0.43	18.53 ± 0.60	17.66 ± 0.84

* Compared with the same indicator from blank nanobubbles, P < 0.05

^#^Compared with the same indicator from BxPC-3 transplanted tumor tissues, P < 0.05

### Localization of targeted nanobubbles in transplanted tumor tissues

**Fig. 8:**
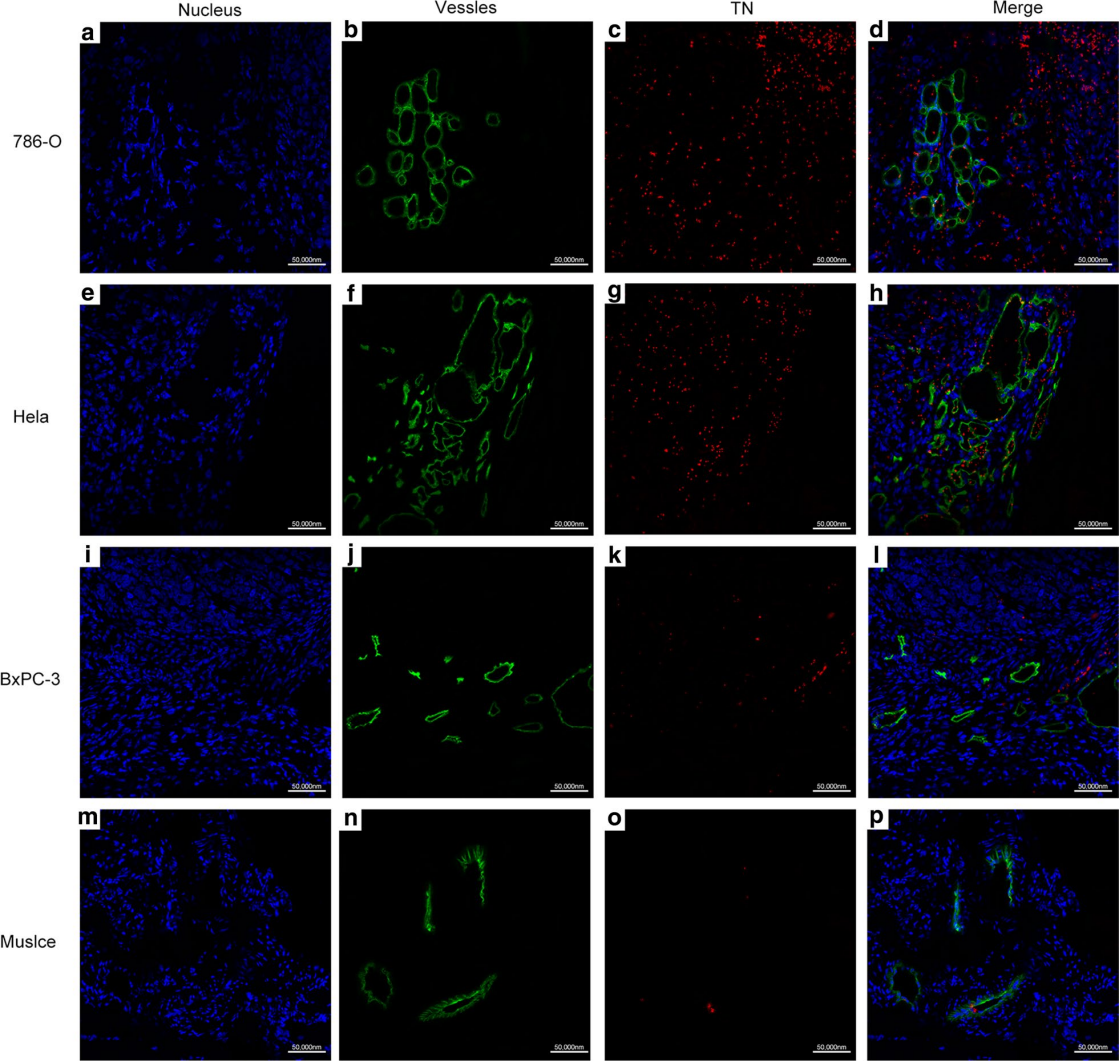
Localization of targeted nanobubbles in nude mice body. **a**–**d** The distribution of targeted nanobubbles in 786-O transplanted tumor tissues. **e**–**h** The distribution of targeted nanobubbles in Hela transplanted tumor tissues. **i**–**l** The distribution of targeted nanobubbles in BxPC-3 transplanted tumor tissues. **m**–**p** The distribution of targeted nanobubbles in the right thigh muscle tissues

Observation of the frozen sections of the transplanted tumor tissues and the right thigh muscle tissues after injection of DiI-labeled targeted nanobubbles, targeted nanobubbles were distributed in tumor blood vessels and tissue spaces outside blood vessels in 786-O, Hela, and BxPC-3 transplanted tumor tissues (Fig. 8a–l). Targeted nanobubbles with intact morphology could occasionally be observed in tissue spaces. While targeted nanobubbles were only present in blood vessels, and no targeted nanobubbles in tissue spaces in the right thigh muscle tissues (Fig. 8m–p). Targeted nanobubbles in 786-O and HeLa transplanted tumor tissue spaces were significantly more than those in BxPC-3 transplanted tumor tissue spaces.

### MVD and CAIX expression in transplanted tumor tissues

**Fig. 9:**
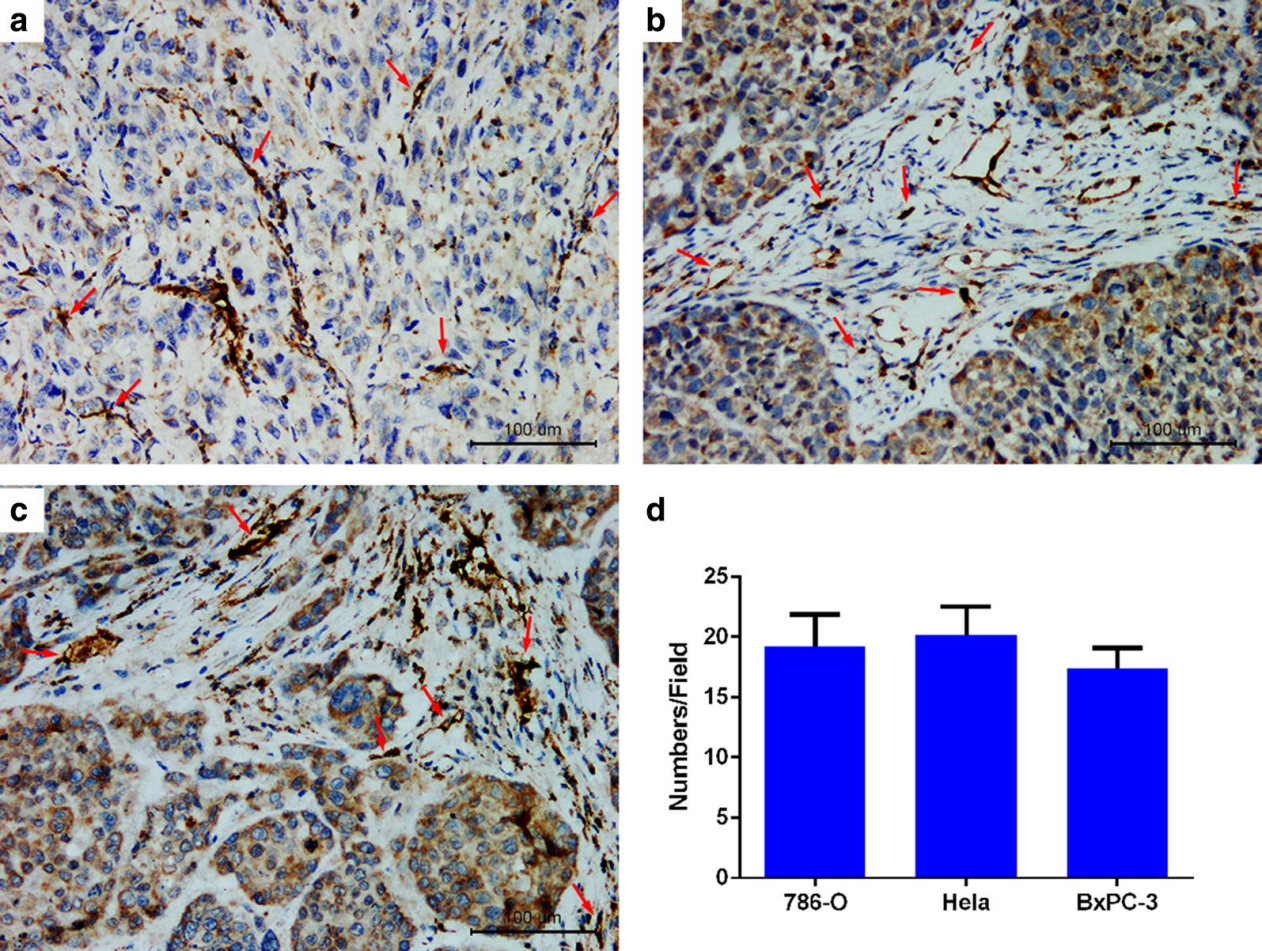
Blood vessels in three types of transplanted tumor tissues. **a** Blood vessels in 786-O transplanted tumor tissues. **b** Blood vessels in Hela transplanted tumor tissues. **c** Blood vessels in BxPC-3 transplanted tumor tissues. **d** MVDs in the three types of transplanted tumor tissues were assessed. Red arrows indicate tumor blood vessels

**Fig. 10:**
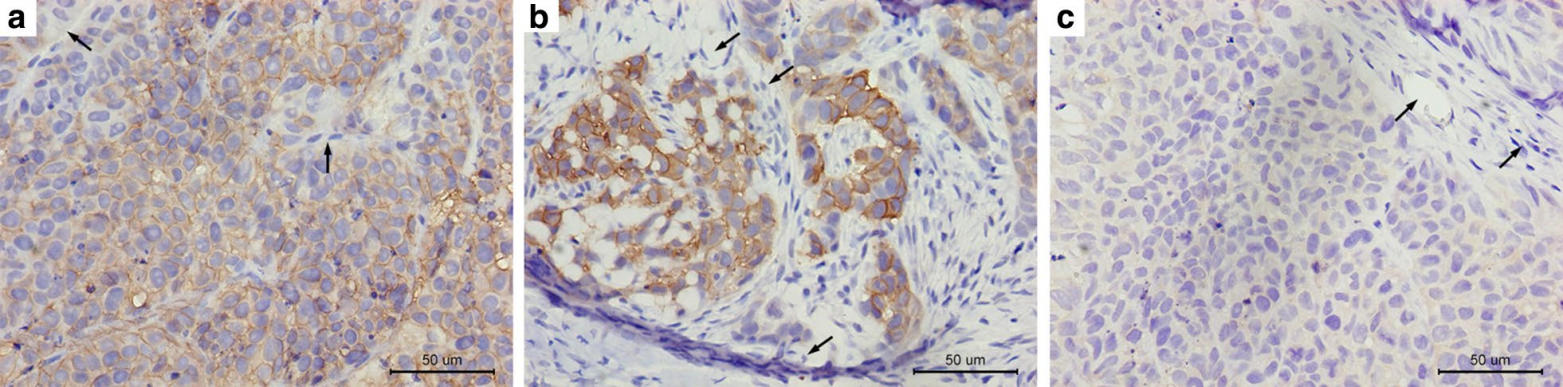
Expression of CAIX in three types of transplanted tumor tissues. **a** Immunohistochemistry on 786-O transplanted tumor tissues. **b** Immunohistochemistry on Hela transplanted tumor tissues. **c** Immunohistochemistry on BxPC-3 transplanted tumor tissues. The yellow–brown areas indicate positive CAIX expression, black arrows indicate tumor stroma

To clarify the reason of the difference in distribution of targeted nanobubbles in the three types of transplanted tumor tissues, we analyzed MVD and CAIX expression in them. Blood vessels that stained dark brown were mainly distributed in tumor stroma that infiltrated into tumor parenchyma in 786-O transplanted tumor tissues and MVD was 19.20 ± 2.68 (Fig. 9a). Blood vessels that stained dark brown were distributed in tumor stroma that wrapped around tumor parenchyma in HeLa and BxPC-3 transplanted tumor tissues and MVDs were 20.20 ± 2.28 and 17.40 ± 1.67, respectively (Fig. 9b, c). While MVDs in the three types of transplanted tumor tissues were not different (Fig. 9d). In 786-O and Hela transplanted tumor tissues, only tumor parenchyma cells (yellow–brown areas) expressed CAIX, and tumor stroma cells (black arrows) and necrotic areas did not express CAIX (Fig. 10a, b), while no BxPC-3 transplanted tumor tissues expressed CAIX (Fig. 10c). Some apparent vascular structures were visible in tumor stroma of the three types of transplanted tumor tissues, which did not express CAIX.

## Discussion

Malignant tumors severely affect human life and health. Early diagnosis and timely treatment contribute greatly to increasing the quality of life and improving the prognosis of cancer patients, especially the common malignant tumors such as cervical cancer and kidney cancer [29, 30]. Due to conventional ultrasound imaging techniques do not reveal the change of molecular expression in malignant tumors prior to the change in anatomical structures, early diagnosis and timely treatment of malignant tumors cannot be truly realized [31]. Through tumor-targeted ultrasound molecular probes (i.e., targeted UCAs), ultrasound molecular imaging provides a novel method for early identification of the change in molecular expression in malignant tumors.

UCAs currently used for molecular imaging of tumors have two main disadvantages. (1) UCAs are mostly at micron scale, so they can only achieve imaging in blood pool and cannot aggregate in tumor tissues to achieve specific imaging of tumor parenchymal cells [32–35]. For example, BR55 that targets vascular endothelial growth factor receptor (VEGFR) has been used in tumor diagnosis [4]. The EPR effect of tumor blood vessels theoretically only allows nanoscale UCAs (e.g., nanobubbles) pass through tumor blood vessels and achieve targeted ultrasound molecular imaging of stroma and parenchymal cells of tumor tissues [36–39]. (2) Targeted UCAs generally aim at only one type of tumor, and only can be used in early diagnosis of one particular malignant tumor, so their clinical application value is limited. For example, we were only able to perform targeted ultrasound imaging of prostate cancer and metastasis and could not perform targeted ultrasound imaging of other tumors in our previous studies [13].

To construct targeted UCAs that specifically aggregate in tumor tissues and achieve ultrasound molecular imaging of a variety of tumors, it is necessary to link ligands that have common specific targets in a variety of tumors to UCAs [40]. Previous studies showed that CAIX is highly expressed on cell membranes of a variety of malignant tumors, including lung, kidney, colon and rectum, breast, cervix, head and neck, and bladder cancer. Due to extracellular proteoglycan domain of CAIX is a specific structure, ligands that target proteoglycan domain of CAIX do not cross-react with other carbonic anhydrase isoenzymes [14].

Polypeptide molecules are ideal ligands in molecular imaging, because their relatively low molecular weights, high selectivity and specificity, strong cell permeability, easily modified without affecting specific characteristics, and low immunogenicity [18, 41, 42]. PGLR-P1 polypeptides specifically bind to CAIX protein, and smaller than other ligand, such as aptamer and monoclonal antibodies. Moreover, there are no current studies on the construction of targeted nanobubbles that carry polypeptides targeting parenchymal cells or targeted ultrasound molecular imaging of many tumors.

Combining the advantages of CAIX, polypeptides and nanobubbles, we used biotin–streptavidin system to construct targeted nanobubbles carrying CAIX polypeptides in the study, whose particle size was only 503.7 ± 78.47 nm. The novel targeted nanobubbles possessed the advantages of homogenous particle size, high stability, and good safety. We used DiI to label lipid membranes of targeted nanobubbles and FITC to modify the polypeptides. Under a LSCM, green fluorescence and red fluorescence could completely overlap, indicating targeted nanobubbles were constructed successfully and the polypeptides evenly distributed on the surfaces of targeted nanobubbles. MTT assay showed that the cytotoxicity of targeted nanobubbles and blank nanobubbles was not significantly different. Targeted nanobubbles carrying CAIX polypeptides specifically aggregated on CAIX-positive tumor cells in vitro at the cellular level.

To analyze ultrasound imaging characteristics of targeted UCAs in vivo is extremely important in ultrasound molecular imaging. Peak time, peak intensity, and duration time of targeted and blank nanobubbles in CAIX-negative tissues (liver, kidney and BxPC-3 transplanted tumor tissues) were not different. While peak intensity and duration time of targeted nanobubbles were significantly better than those of blank nanobubbles in CAIX-positive tissues (786-O and Hela transplanted tumor tissues), and also significantly better than those of targeted nanobubbles in CAIX-negative tissues (BxPC-3 transplanted tumor tissues). The reason probably is that targeted nanobubbles carrying CAIX polypeptides actively aggregated in CAIX-positive tumor tissues, so the number of targeted nanobubbles in the cross-sections of CAIX-positive transplanted tumor tissues was significantly more than others, including the number of blank nanobubbles in CAIX-positive transplanted tumor tissues and the number of targeted nanobubbles in CAIX-negative transplanted tumor tissues. Combined with the feature of nanobubbles aggregation imaging, the imaging effect of targeted nanobubbles was better than that of blank nanobubbles in CAIX-positive transplanted tumor tissues, also better than that of targeted nanobubbles in CAIX-negative transplanted tumor tissues, which was consistent with the results of Cai et al. [9, 24, 43–45].

The basis of ultrasound molecular imaging is that targeted nanobubbles penetrate blood vessels and enter into transplanted tumors tissue spaces, so that to determine the distribution of targeted nanobubbles in different types of transplanted tumor tissues is important. We found targeted nanobubbles could pass through tumor blood vessels and reach the three types of transplanted tumor tissue spaces, while could not be distributed in the right thigh muscle tissue spaces. Meanwhile, we found that a number of targeted nanobubbles were distributed in CAIX-positive transplanted tumor tissues (786-O and HeLa transplanted tumor tissues) and little targeted nanobubbles were distributed in CAIX-negative transplanted tumor tissues (BxPC-3 transplanted tumor tissues).

Considering tumor blood vessels play an important role in CEUS imaging, we analyzed MVD of the transplanted tumor tissues [43]. However, MVDs were not significant different in the three types of transplanted tumor tissues, so tumor blood vessel was not the reason of the imaging differences of targeted nanobubbles in the three types of transplanted tumor tissues. At last, we confirmed 786-O and HeLa transplanted tumor tissues expressed CAIX, while BxPC-3 transplanted tumor tissues did not express CAIX. Therefore, the expression of CAIX resulted in the imaging difference of targeted nanobubbles in the three types of transplanted tumor tissues, which could promote targeted nanobubbles to aggregate in CAIX-positive transplanted tumor tissues and then enhanced transplanted tumor imaging.

Due to targeted nanobubbles carrying CAIX polypeptides could pass through tumor blood vessels and specifically enhanced ultrasound molecular imaging in CAIX-positive transplanted tumor tissues, ultrasound molecular imaging could find the change of CAIX expression prior to the change in anatomical structures and achieve early diagnosis of many solid tumors derived from various organs. Though some studies have confirmed that the binding ability and imaging effect of blank microbubbles without carrying ligand were nearly identical with those of scrambled microbubbles carrying nonsense ligand, and the two kinds of microbubbles had a poorer capacity than those of targeted microbubbles carrying specific ligand in positive expression tissues, scrambled nanobubbles carrying nonsense polypeptides as the negative control may be more complete [46, 47].

## Conclusions

In summary, we used biotin–streptavidin system to construct targeted nanobubbles carrying CAIX polypeptides, which possessed the advantage of excellent penetration ability and strong specificity. In addition, dual-fluorescence confirmed the polypeptides evenly distributed on the surfaces of targeted nanobubbles and immunofluorescence confirmed targeted nanobubbles specifically aggregated in CAIX-positive tumor tissues. This study not only provides a novel ultrasound molecular probe for targeted ultrasound molecular imaging of a variety of tumors derived from various organs, but also further describes targeted nanobubbles could pass through tumor blood vessels to enter in tumor tissue spaces and clarifies the imaging differences of targeted nanobubbles in different types of transplanted tumor tissues.

## Additional file

**Additional file 1.** Mass spectrogram of polypeptides. a Mass spectrogram of the unmodified polypeptides. b Mass spectrogram of the FITC-modified polypeptides.

## Abbreviations

CAIX: carbonic anhydrase IX
CE-MRI: contrast-enhanced magnetic resonance imaging
CE-CT: contrast-enhanced computed tomography
CEUS: contrast-enhanced ultrasound
UCAs: ultrasound contrast agents
EPR: enhanced permeability and retention
PBS: phosphate-buffered saline
TN: targeted nanobubbles
BN: blank nanobubbles
DMSO: dimethyl sulphoxide
BSA: bovine serum albumin
LSCM: laser scanning confocal microscope
MVD: microvessel density
VEGFR: vascular endothelial growth factor receptor
