# Supplementary material for: Construction of ultrasonic nanobubbles carrying CAIX polypeptides to target carcinoma cells derived from various organs
Source: J Nanobiotechnology. 2017 Sep 29;15:63. doi: 10.1186/s12951-017-0307-0 (PMC5622542; doi:10.1186/s12951-017-0307-0)
Supplement: Supplementary file 1 — Additional file 1. Mass spectrogram of polypeptides. a Mass spectrogram of the unmodified polypeptides. b Mass spectrogram of the FITC-modified polypeptides. [file 12951_2017_307_MOESM1_ESM.pdf]

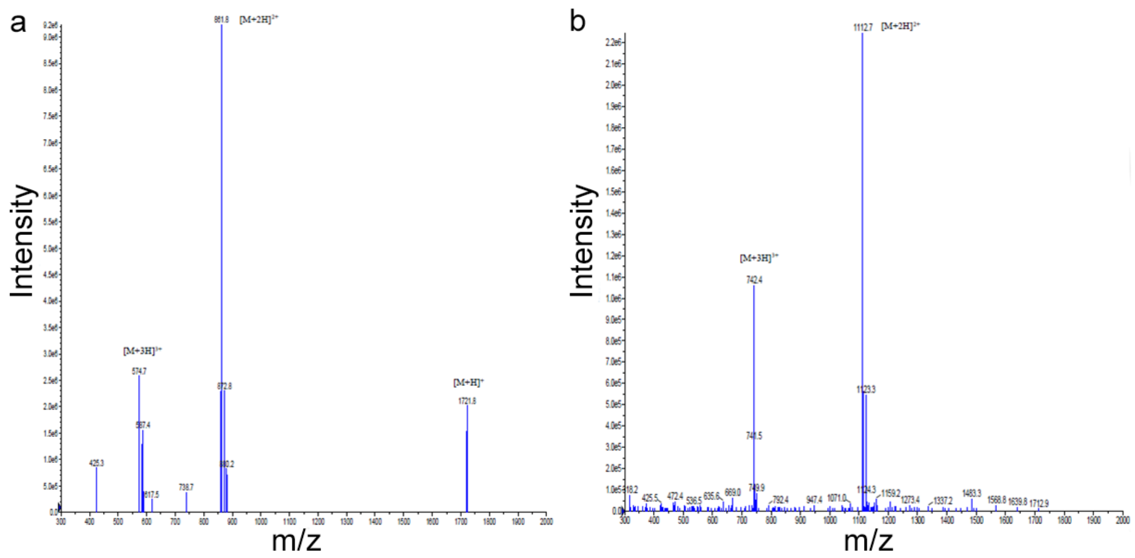

Additional file 1 Mass spectrogram of polypeptides. **a** Mass spectrogram of the unmodified polypeptides. **b** Mass spectrogram of the FITC-modified polypeptides.
